# Use of Recombinant Tobacco Mosaic Virus To Achieve RNA Interference in Plants against the Citrus Mealybug, *Planococcus citri* (Hemiptera: Pseudococcidae)

**DOI:** 10.1371/journal.pone.0073657

**Published:** 2013-09-09

**Authors:** Arif Muhammad Khan, Muhammad Ashfaq, Zsofia Kiss, Azhar Abbas Khan, Shahid Mansoor, Bryce W. Falk

**Affiliations:** 1 National Institute for Biotechnology and Genetic Engineering (NIBGE), Faisalabad, Pakistan; 2 Pakistan Institute of Engineering and Applied Sciences (PIEAS), Islamabad, Pakistan; 3 Department of Plant Pathology, University of California Davis, Davis, California, United States of America; 4 Biodiversity Institute of Ontario, University of Guelph, Guelph, Ontario, Canada; 5 Department of Entomology, University of Sargodha, Sargodha, Pakistan; United States Department of Agriculture, United States of America

## Abstract

The citrus mealybug, *Planococcus citri*, is an important plant pest with a very broad plant host range. *P. citri* is a phloem feeder and loss of plant vigor and stunting are characteristic symptoms induced on a range of host plants, but *P. citri* also reduces fruit quality and causes fruit drop leading to significant yield reductions. Better strategies for managing this pest are greatly needed. RNA interference (RNAi) is an emerging tool for functional genomics studies and is being investigated as a practical tool for highly targeted insect control. Here we investigated whether RNAi effects can be induced in *P. citri* and whether candidate mRNAs could be identified as possible targets for RNAi-based *P. citri* control. RNAi effects were induced in *P. citri*, as demonstrated by specific target reductions of *P. citri actin, chitin synthase* 1 and *V-ATPase* mRNAs after injection of the corresponding specific double-stranded RNA inducers. We also used recombinant *Tobacco mosaic virus* (TMV) to express these RNAi effectors in *Nicotiana benthamiana* plants. We found that *P. citri* showed lower fecundity and pronounced death of crawlers after feeding on recombinant TMV-infected plants. Taken together, our data show that *actin, chitin synthase* 1 and *V-ATPase* mRNAs are potential targets for RNAi against *P. citri*, and that recombinant TMV is an effective tool for evaluating candidate RNAi effectors in plants.

## Introduction

The citrus mealybug, *Planococcus citri* (Hemiptera: Pseudococcidae), is a serious pest of citrus, nursery plants, grapes and is occasionally considered a serious pest of greenhouse crops [1]. On citrus, *P. citri* damages plants by feeding on roots, bark, foliage and fruit [2]. During feeding *P. citri* injects toxic saliva into plants and extracts phloem sap, and induces symptoms including defoliation, fruit discoloration and fruit drop [2]. In addition, *P. citri* secretes copious amounts of honeydew leading to the development of sooty mold which also leads to reduced fruit quality and lowered plant vigor through the loss of photosynthetic capacity.

RNA interference (RNAi) is a natural gene regulation and anti-viral defense found in eukaryotes. RNAi has not only been used to study gene function [3]–[5], but it has shown as a potential strategy for controlling insect pests [6]–[9]. Double-stranded (ds) and short-interfering RNAs (siRNAs) both can induce RNAi effects in insects [10], resulting in degradation of the target mRNA. If the target mRNA is critical for insect development, severe phenotypic effects or even death may result [10], [11]. RNAi effects have been achieved for insects by introducing specific ds- and siRNAs into the insects body by microinjection [12], [13], by feeding [14]–[20] or even by exogenous applications [21]. For plant-feeding insects, using transgenic plants engineered to express dsRNAs corresponding to insect target mRNAs has also shown potential [7], [17]. Thus, for practical RNAi application it is critical to identify targets which are vital for the insect and which could successfully be suppressed through RNAi.

RNAi effects have been demonstrated in many insects including fruit flies (*Drosophila melanogaster*), flour beetles (*T. castaneum*), pea aphids (*Acyrthosiphon pisum*), and tobacco hornworms (*Manduca sexta*) that were all selectively killed when fed species-specific dsRNAs targeting *V-ATPase* mRNAs [10], [18]. Moreover, other studies have shown that using a transient plant-mediated RNAi delivery system for pea aphid (*A. pisum*) against the *rak*1 gene that is predominantly expressed in the gut, results in great reduction of the *rak1* mRNA levels. This approach was further extended to the *C002* gene that is expressed in the salivary glands and has also shown reduction in the mRNA levels of *C002* [16]. In another study on potato/tomato psyllids (*Bactericerca cockerelli*) gut-specific mRNAs were successfully targeted through microinjection and oral acquisition of interfering RNAs [10]. Recently, RNAi effects were induced in the Glassy-winged sharpshooter (*Homalodisca vitripennis*) by microinjection of interfering RNAs corresponding to *actin* mRNAs, thus reducing the mRNA levels and causing mortality in the injected *H. vitripennis* [11]. Furthermore, RNAi has been used successfully in transgenic corn plants against *Diabrotica virgifera virgifera* [7]. These transgenic plants showed a reduction in damage caused by *D. virgifera virgifera* suggesting that the RNAi pathway can be exploited to control insect pests via *in planta* expression of interfering RNAs. Earlier studies also showed reductions of *CYP6AE14* transcripts in the *D. virgifera virgifera* midgut after feeding the larvae on plant material expressing dsRNAs specific to *CYP6AE14*, and this also resulted in a severe retardation of larval growth [17].

By contrast RNAi effects were not successfully achieved for several other insects. In *Bombyx mori*, injection of dsRNA targeting the ecdysone receptor mRNA or other targets in the epidermis did not result in detectable RNAi effects [22]. Similarly, a series of unsuccessful attempts through feeding dsRNAs were reported for *Helicoverpa armigera* and *Spodoptera frugiperda* [22], while two other independent studies showed efficient targeting of mRNAs expressed in the *S. frugiperda* midgut and brain after feeding dsRNAs [23], [24]. Interestingly, while feeding long dsRNAs to *H. armigera* larvae was not very successful [22], good RNAi effects were achieved using custom-designed siRNAs [25], suggesting different efficiencies for short siRNAs vs. long dsRNAs, or at least their specific sequences, in these species. In general, there seems to be a positive correlation between amount of dsRNA and the degree of RNAi effects [22]. Interestingly, for *Bicyclus anynana, Chrysodeixis includes* and *Spodoptera littoralis* [22], [26], [27] even higher amounts of dsRNA (>1 mg per mg of tissue) did not result in detectable RNAi effects. This variation in responses to RNAi by different species may be due to variation in delivery methods, dose of effector RNAs introduced, RNAi targets or by differences in species with respect to their susceptibility to RNAi [22].

The above studies suggest that RNAi approaches could potentially be used as a tailor-made insecticide for at least some insects, but the correct target, effector RNA, and means of effector delivery must be identified in order to achieve desired RNAi effects. It is also likely that in insects, a continuous supply of effector RNAs may be required. Most insects appear to lack RdRp (RNA dependent RNA Polymerase) [6], [28]–[30] which in plants serves to amplify the RNAi effects thereby producing a continuous supply of effector RNAi molecules [31]. Thus, transgenic plants engineered to express a continuous supply of RNAi effectors are desirable. However, all mRNAs are not equal targets, and assessing potential effector RNAs via transgenic plants is a tedious, time consuming and expensive effort [32]. Recombinant plant virus vectors engineered to express the desired insect effector RNAs in plants provide a potential alternative approach that circumvents many of the above problems seen for transgenic plants [32]. One such virus is *Tobacco mosaic virus* (TMV) [33]. TMV has a wide plant host range, it is easily engineered, mechanically transmissible from plant to plant, and plants respond quickly to virus infection by producing TMV-specific siRNAs. Then if TMV can be engineered to contain sequences of the desired mRNA target, siRNAs towards this target will be generated. Furthermore, TMV replicates through dsRNA intermediates, and thus plants infected with recombinant TMVs produce at least two forms of RNAi effectors, specific siRNAs, and replicative form dsRNAs.

There is no information available on RNAi activity in *P. citri*. In this study we first determined if RNAi effects could be induced in *P. citri* and then we attempted to identify potential mRNA targets that might offer opportunities for *P. citri* control using RNAi based approaches. We used *in vitro* synthesized dsRNAs and recombinant-TMV infected plants and demonstrated the effectiveness of *P. citri* RNAi targets via both of these assays.

## Materials and Methods

### Insect rearing

Insects were collected from naturally infested *Vitis vinifera* plants on the U. C. Davis campus with the permission of greenhouse manager Mr. Steven Silva. *P. citri* were reared on *Nicotiana tabacum* and *Solanum lycopersicum* plants under greenhouse conditions at 24–28°C. Second instar *P. citri* nymphs were used in feeding and 3^rd^ instars for injection bioassay studies to evaluate the RNAi effects.

### RNA extractions

RNA was extracted from whole insects or insect body parts using Trizol reagent following the manufacturer's protocol (Ambion, Cat. No. 15596018). RNAs were precipitated using two volumes of ethanol and 1/10 volume of 3 M sodium acetate. RNA pellets were washed with 70% ethanol, and re-suspended in DEPC-treated diH_2_O.

### PCR amplification and analysis of *P. citri* sequences

Published *chitin synthase* 1 (CHS1) sequences of *Aedes aegypti* (XM_001662150.1), *Anopheles gambiae* (XM_321951.2), *Ostrinia furnacalis* (EU258740.1), *Culex quinquefasciatus* (XM_001864559.1), *Plutella xylostella* (AB271784.1), *Nasonia vitripennis* (XM_001602131.2) and *Tribolium castaneum* (NM_001039403.1) were aligned using ClastalW. Universal forward and reverse primers (Table 1) were designed from conserved regions to amplify a 507bp fragment of CHS1 from *P. citri*. V-ATPase primers were designed in the same fashion from the conserved region of the gene by aligning published V-ATPase sequences of *B. mori* (NM_001098359.1), *M. sexta* (X64233.1), *Apis mellifera* (XM_623492.3), *T. castaneum* (XM_971095.2), *A. aegypti* (AF008922.1), *Aedes albopictus* (AY864912.1) and *N. vitripennis* (XM_003426538.1). Actin gene sequence was isolated using primers designed from P. solenopsis actin gene sequence (unpublished data) (Table 1).

**Table 1:** Primers used for RT-PCR and cDNA synthesis.

SequenceName	Primer Name	Primer sequence 5′ to 3′	Product Size (bp)	RT-PCR Conditions
Chitin synthase	CSFCSR	GGCTCTGGTCCTATGGTGTGGTATCA aGCCACRAAAGCACCCACCAACATAAGGAA	507	94°C for 3min, 35cycles of 94°C for 30Sec, 48°C for 45sec, 72°C for 1min and finally 72°C for 10 min
V-ATPase	VATPase F1VATPase R1	aGGCYACYATHCAGGTATA^a^TCVARMCCCCAGAACAC	1122	94°C for 3min, 35cycles of 94°C for 30Sec, 50°C for 45sec, 72°C for 1min and finally 72°C for 10 min
Actin	MbActinSalFMbActinSmaR	bGTCGACTCCGGTGATGGTGTATCTCA^c^CCCGGGATCACGGACGATTTCTCGTT	171	94°C for 3min, 35cycles of 94°C for 30Sec, 50°C for 45sec, 72°C for 1min and finally 72°C for 10 min
PJL36	PJL36 leftPJL36 Right	AGATCTTACAGTATCACTACTCC GTACGCACCACGTGTGATTACGG	100	94°C for 3min, 35cycles of 94°C for 30Sec, 55°C for 45sec, 72°C for 1min and finally 72°C for 10 min

a The following degeneracy codes are used in the primer sequences:

R = G/A, Y = C/T, H = A/C/T, V = A/C/G, M = A/C.

b Underlined nucleotide sequence is the *Sal* 1 restriction enzyme recognition sequence, added to facilitate cloning.

c Underlined sequence is the *Sma* 1 restriction enzyme recognition sequence, added to facilitate cloning.

First strand cDNA was synthesized using the superscript cDNA synthesis kit and with an oligo (dT)-primer using 500 ng RNA and following manufacturer's instruction (Invitrogen, Cat. No. 11904–018). Briefly RNA was incubated with oligo (dT) primer at 65°C for 5 min. A 10 µl reaction mixture (10X RT buffer 2 µl, 25 mM MgCl_2_ 4 µl, 0.1 M DTT 2 µl, RNAse out (40 U/µl) 1 µl and SuperScript II 1 µl) was added and samples were incubated at 42°C for 50 min followed by 10 min incubation at 70°C. The cDNA (200 ng/reaction) was used as template for amplification through PCR using GoTaq Flexi DNA polymerase (Promega Corp., Madison, WI, USA Cat. No. M8291) and universal primers at the PCR conditions given (Table 1).

PCR products were analyzed by agarose gel electrophoresis, purified using the ZymoClean PCR cleaning kit (Zymo Corp., Irvine, CA, USA Cat. No. D4006), and subsequently cloned into pGEM-T Easy (Promega Corp., Madison, WI, USA Cat. No. A1360). Sequencing was done by the DNA sequencing facility at UC Davis using M13 forward and reverse primers. The resulting sequences were confirmed by protein translations and BLAST searches (http://www.ncbi.nlm.nih.gov) and gene-specific primers were designed. A second round of PCRs was performed to amplify the cDNAs to be used to generate dsRNAs (Table 2). The PCR products were sequenced and sequences were submitted to GenBank (NCBI) with accession numbers JX443530 and JX443529 of *CHS*1 and *V-ATPase* gene fragments, respectively. The actin sequence was submitted to European Nucleotide Archive (EMBL) with accession number HF952554.

**Table 2:** Primers used for RT-PCR of RNAi targets.

SequenceName	Primer Name	Primer sequence 5′ to 3′	Product Size (bp)	RT-PCR Conditions
Chitin synthase	PcCHSSalFPcCHSSmaR	aCGTCGAC CGTTGGCTGTGCACATTACT bCCCGGG AAGCACCCCACCAACATAAG	290	94°C for 3min, 35cycles of 94°C for 30Sec, 55°C for 45sec, 72°C for 1min and finally 72°C for 10 min
V-ATPase	PCVATPSalFPCVATPSmaR	aCGTCGACAAGGTTGGCAGCCACATAAC^b^CCCGGGCGAAAGCTCCTGGTATAGCG	332	94°C for 3min, 35cycles of 94°C for 30Sec, 57°C for 45sec, 72°C for 1min and finally 72°C for 10 min
Actin	PCActinSalFPCActinSmaR	aCGTCGACTCCGGTGATGGTGTATCTCA^b^CCCGGGATTTCTCGTTCGGCAGTTGT	168	94°C for 3min, 35cycles of 94°C for 30Sec, 55°C for 45sec, 72°C for 1min and finally 72°C for 10 min
GFP	PacI-GFP leftJAL34	cCGGTTAATTAAATGGCTAGCAAAGGAGAAGAAC^d^TTTGCGGCCGCTTATTTGTAGAGCTCATCCATG	742	94°C for 3min, 35cycles of 94°C for 30Sec, 55°C for 45sec, 72°C for 1min and finally 72°C for 10 min

a Underlined nucleotide sequence is the *Sal* 1 restriction enzyme recognition sequence, added to facilitate cloning.

b Underlined sequence is the *Sma* 1 restriction enzyme recognition sequence, added to facilitate cloning.

c Underlined nucleotide sequence is the *Pac* 1 restriction enzyme recognition sequence, added to facilitate cloning.

d Underlined nucleotide sequence is the *Not* 1 restriction enzyme recognition sequence, added to facilitate cloning.

### DsRNA synthesis and microinjection of *P. citri*

The RNAi target regions of *CHS*1 and *V-ATPase* mRNAs used here were selected using the SnapDragon dsRNA design software (http://www.flyrnai.org/cgi-bin/RNAi_find_primers.pl). Regions with sufficient nucleotide divergence from those of other insect species were selected to avoid potential off-target effects. The T7 promoter sequence was added to the 5′ ends of both forward and reverse primers (Table 3), PCR products were amplified from cDNA and purified using the ZymoClean PCR cleaning kit (Zymo Corp., Irvine, CA, USA Cat. No: D4006). Purified PCR products were used as templates to synthesize dsRNA using T7 RNA polymerase and the MEGAscript RNAi Kit as per manufacturer's instructions (Ambion, Austin, TX, USA Cat. No. AM1626). The dsRNAs were treated with DNAse and RNAse at 37°C for 30 min following the manufacturer's protocol, and electrophoresed on an agarose gel to assess dsRNA quality.

**Table 3:** Primers used for *in vitro* dsRNA synthesis.

Sequence Name	Primer Name	aPrimer sequence 5′ to 3′	Product Size (bp)
Chitin Synthase	PCCHST7FPCCHST7R	GGATCCTAATACGACTCACTATAGGG CGTTGGCTGTGCACATTACT GGATCCTAATACGACTCACTATAGGG AAGCACCCCACCAACATAAG	342
V-ATPase	PCVATPT7FPCVATPT7R	GGATCCTAATACGACTCACTATAGGG AAGGTTGGCAGCCACATAAC GGATCCTAATACGACTCACTATAGGG CGAAAGCTCCTGGTATAGCG	384
Actin	PCActinT7FPCActinT7R	GGATCCTAATACGACTCACTATAGGG TCCGGTGATGGTGTATCTCA GGATCCTAATACGACTCACTATAGGG ATTTCTCGTTCGGCAGTTGT	220
GFP	GFPFGFPR	bCTAATACGACTCACTATAGG *GCGGCCGC*ACGCGTGCTGAAGTCAAGTT^b^CTAATACGACTCACTATAGG *GCGGCCGCC*TTTTCGTTGGGATCTTTCG	321

a Underlined nucleotide sequence is the T7 RNA polymerase promoter sequence. A pair of gene specific primers with the T7 promoter sequence at the 5′ ends of both primers was used to amplify the desired templates. Amplified PCR products were used as templates for *in vitro* dsRNA synthesis.

b Italic nucleotide sequence is the *Not* 1 restriction enzyme recognition sequence.

To assess if RNAi effects could be induced in *P. citri*, individual mealybug third instar nymphs were injected with 200 nl of dsRNA by using a micromanipulator and microinjector (http://www.tritechresearch.com/MINJ-PD.html). For microinjection, insects were placed individually on a 2% agarose gel to immobilize them, and were injected with 200 nl (500 ng/µL) of dsRNA using fine glass needles and the micromanipulator/miroinjector. Injected insects were moved back to fresh tobacco leaves at 24–26^o^C for post-injection observations and procedures. RNA was extracted from injected *P. citri* at 48 h, 72 h, 96 h and 150 h post-injection and qRT-PCR was performed to assess target mRNA amounts.

### Cloning in TMV

Cloned sequences for the mRNA targets of interest (*actin, CHS*1 and *V-ATPase*) were excised from pGEM-T Easy by digesting with *Not*I, and ligated into the *Not*I-digested TMV-based vector, pJL36 [33]. Positive clones were screened first by restriction digestion and then by nucleotide sequence analysis to confirm the insert sequence orientation. Confirmed clones were transformed into *Agrobacterium tumefaciens* (GV3101 strain) for inoculation to *N. benthamiana* plants. Plants were inoculated by agro infiltration with the recombinant TMV-based vector following the protocol as described previously [34]. RT-PCR was performed using insect gene specific primers to check the presence of insect target mRNA in the inoculated plants. The pJL24 TMV-based vector has been engineered to express green fluorescent protein (GFP) was used as a control. Reverse transcriptase polymerase chain reaction (RT-PCR) was used to verify retention of the inserted sequence by the recombinant TMVs in infected plants. RNA was extracted from upper symptomatic leaves of inoculated plants, and primers from the TMV vector sequence flanking the insert were used for RT-PCR (Table 1).

### SiRNA detection

Seven days post inoculation total RNAs were extracted from plant leaves using Trizol reagent following the manufacturer's instructions. The small RNA fraction was isolated from total RNA using 50% PEG 8000 precipitation. Small RNAs were electrophoresed on a 15% 8 M urea polyacrylamide gel with microRNA markers (New England BioLabs, Inc, Ipswich, MA, USA Cat. No. N2102S). RNAs were transferred to Hybond-NX Amersham membrane (GE Healthcare, Piscata way, NJ Cat. No. RPN203T) using a semi dry blotter as described [35]. A ^32^P-UTP labeled probe was prepared by *in vitro* transcription using the Maxiscript T7 kit (Ambion, Austin, TX, USA Cat. No. AM1312) as described [11] and the resulting probe was fragmented by adding carbonation buffer (20 mM Na_2_CO_3_ and 80mM NaHCO_3_). Hybridization was done overnight in Ultrahyb-oligo hybridization buffer (Ambion, Austin, TX, USA Cat. No. AM8669) at 42°C followed by two washes in Northern Max Low Stringency Wash Solution (Ambion, Austin, TX, USA Cat. No. AM8673) for 15 minutes at 42°C. The blot was drained and then exposed to X-Ray film (F-BX810, Phenix) at −80°C for 72 hrs.

### Insect bioassays and Quantitative Real-time PCR (qRT-PCR) analyses

At seven days post inoculation (dpi), ten 2^nd^ instar mealybugs were placed on inoculated and upper leaves of *N. benthamiana* plants. Data were recorded on mortality, insect growth, ovisac production, crawler emergence and their survival. RNA was extracted from individual mealybugs after feeding for 12 days on the respective TMV-infected plants, and four days post dsRNA-injection. cDNA was synthesized using 500 ng total RNA and oligo(dT) primer. Quantitative real time polymerase chain reaction (qRT-PCR) was performed using gene specific primers with the 18S rRNA as the internal control (Table 4) using Fast SYBR Green Master Mix (Applied Biosystem Cat. No. 4385610) and comparative mRNA amounts were analyzed by 2^–ΔΔCT^ method [10] using ABI 7500 system software v2.0.1. The reaction mixture contained 8.5 µl SYBR Green Master Mix, 0.5 µl 10 µM forward primer, 0.5 µl 10 µM reverse primer and 1 µl cDNA template in a final reaction volume of 20 µl. Forty cycles of PCR were performed for 3 sec at 95^o^C and 30 sec at 60^o^C. The ΔCt value for individual samples were obtained by subtracting average Ct (cycle threshold) value for 18S rRNA from average Ct value for the target RNA. One of the samples in control groups (elution buffer (EB) injected in dsRNA microinjection experiments and GFP dsRNA or siRNA-fed mealybugs in feeding experiments) was selected as a reference sample. The ΔΔCt value for individual samples was obtained by subtracting ΔCt value for the reference sample from the ΔCt value of the test sample. The relative expression of target RNA in comparison to 18S RNA level in each sample is represented by the 2^–ΔΔCt^ value. The mean 2^–ΔΔCt^ value of all samples was divided with the mean 2^–ΔΔCt^ value of the control sample to make easy comparison between the groups. The average value and standard deviation value (SD value) of the 2^–ΔΔCt^ values in treatment and control groups were calculated separately, and the student's t-test was performed between treated and control groups to see the difference between the groups.

**Table 4:** Primers used for qRT-PCR.

Sequence Name	Primer Name	Primer sequence 5′ to 3′
Chitin synthase	PCCHSRTFPCCHSRTR	CACAAAATCTCAAGAAGCTCGTCAT AGTAATGTGCACAGCCAACGAT
V-ATPase	PCATPRTFPCATPRTF	CCGTCGGTGATCCTGTTTTG ACCGGGACCCAATTCGA
18S	PC18SRTFPC18SRTR	TCGGAAGGAACGCTTTTATTAGA ACGCCCGAAAGCGAAAC
GFP	GFP qPCR FGFP qPCR R	CCTGTCCTTTTACCAGACAACCA CACGCTTTTCGTTGGGATCT

Primers for qRT-PCR studies were designed from target sequences using Primer Express 3.0 software and the best primers that did not produce secondary structures or primer dimers were selected. These primers targeted sequences flanking the input dsRNA sequences.

### Statistical Analysis

Experimental data were analyzed to test the difference among treatments at a significance level of 0.05 and 0.01. Analysis of variance (ANOVA) was performed using Statistix 8.1. The homogenous groups are shown with similar letters based on least significant difference (LSD) values. Mortality was corrected using Abbot's formula [36].

## Results

### Sequence analysis

We successfully amplified and sequenced 507bp of *CHS*1, 1120bp of *V-ATPase* and 164bp of *actin* (ACTB) mRNAs from *P. citri*. To determine the sequence similarities of the three sequences from *P. citri* with those from other insect species, we performed BLAST analysis. The *CHS*1 sequence showed a maximum (87%) nucleotide identity with that of *P. solenopsis* (unpublished data) and a minimum (74%) with that of *T. castaneum* (Table S1 in Tables S1). *V-ATPase* sequence showed a maximum (83%) identity with that of *P. solenopsis* (unpublished data) and a minimum (76%) with that of *A. albopictus* (Table S2in Tables S1). BLAST search results of *actin* showed a maximum identity of 88% with that of *P. solenopsis* (unpublished data) and a minimum (76%) with that of *Liposcelis entomophila* (Table S3 in Tables S1). The BLAST search results validated the fidelity of the sequences amplified in our study.

### RNAi induction by microinjection

**Figure 1:**
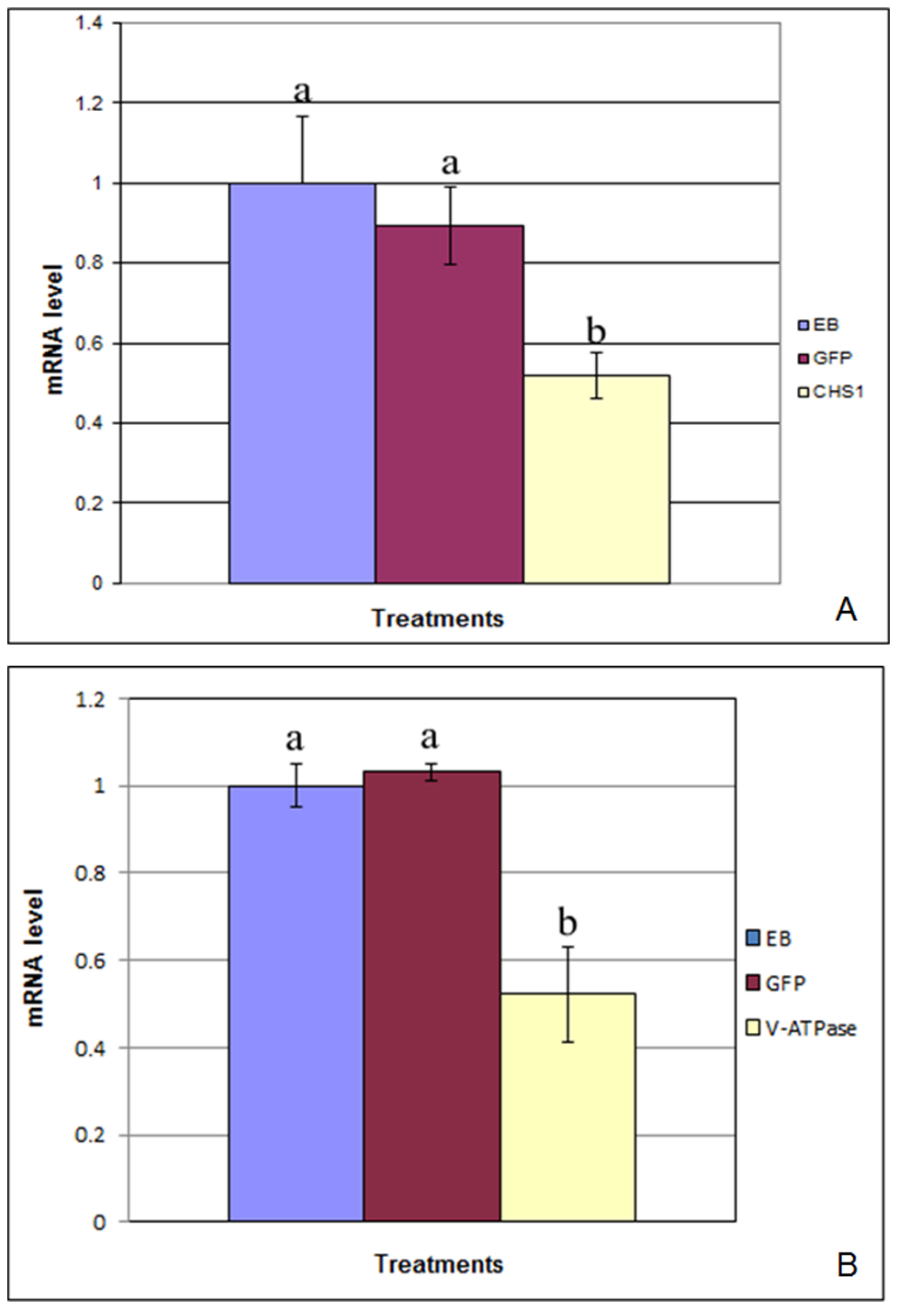
Relative mRNA levels of *CHS*1 (A) and *V-ATPase* (B) following microinjection of specific dsRNAs into third instar *P. citri*. qRT-PCR results of *CHS*1 (A) and *V-ATPase* (B) mRNAs from *P. citri* following microinjection of elution buffer (EB), green fluorescent protein (GFP), *CHS*1 or *V-ATPase* dsRNAs. Total RNA was extracted 4 days post injection and used for cDNA synthesis. qRT-PCR was performed using primers described in Table 3. The *P. citri* 18S ribosomal RNA was used as an endogenous control in all experiments. Calculation of mRNA levels between control and treated groups was done by the comparative CT or 2^–ΔΔ^Ct method and analyzed by ANOVA using statistix 8.1 software. Numbers followed by the same letter are not different at p<0.01 (LSD 0.08). All experiments were performed twice and data were used collectively.

In our experiments there was a significant (>40%) reduction of the target mRNAs in *P. citri* injected with effector dsRNAs as compared to insects injected with elution buffer (EB) or the control GFP dsRNA (Fig. 1A, B). Statistical analysis showed significant differences between samples injected with EB (control) and *V-ATPase* dsRNA, or those injected with GFP dsRNA and *V-ATPase* dsRNA showing an LSD value of 0.08 (P<0.01). Similarly there were significant differences between samples injected with EB and *CHS*1 dsRNA, or GFP dsRNA and *CHS*1 dsRNA showing an LSD value of 0.10 (P<0.01). There was no significant difference between samples injected with EB or GFP dsRNA.

### Recombinant TMV inoculated plants contain *P. citri* RNAs

**Figure 2:**
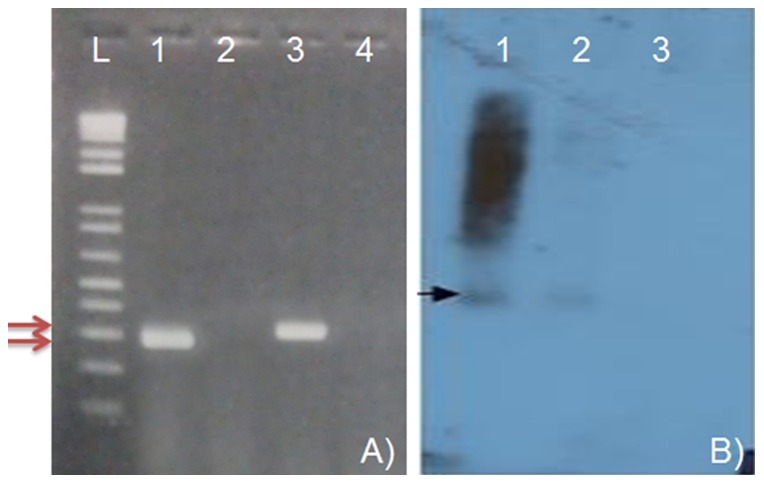
Detection of *P. citri CHS*1 and *V-ATPase* mRNAs (A) and accumulation of RNAi-induced siRNAs (B) in *N. benthamiana* plants after TMV-CHS1 inoculation. Plants were inoculated with recombinant TMV containing *P. citri CHS*1 (TMV-CHS1-S) and *V-ATPase* (TMV-V-ATPase-S). At 7 days post inoculation, total and small RNAs were isolated from infected plants and analyzed by RT-PCR and siRNA northern blot hybridization, respectively. A) One step RT-PCR was performed using total RNA as a template and *CHS*1 and *V-ATPase* primers and products analyzed on the gel. Lane L: 1Kb plus DNA ladder, Lane 1: *CHS*1 primers and RNA of TMV-CHS1-S inoculated *N. benthamiana* plants; Lane 2: *CHS*1 primers and RNA of TMV inoculated plant; Lane 3: *V-ATPase* primers and RNA of TMV-V-ATPase-S inoculated plant and Lane 4: *V-ATPase* primers and RNA of TMV inoculated plant. B) Small RNAs were separated by PAGE in 15% acrylamide, 8 M urea gels and processed for northern blot hybridization using ^32^P-UTP-labeled negative strand *P.citri CHS*1 transcript as a probe. Lane 1: TMV-CHS1-S inoculated plant; Lane 2: TMV-CHS1-AS iinoculated plant; Lane 3: TMV-inoculated plant.

In order to elucidate whether *P. citri* effector RNAs were produced in plants after inoculation using the recombinant TMVs, we first confirmed the presence of the *actin, CHS*1 and *V-ATPase* RNAs by RT-PCR. These plants tested positive by RT-PCR (Fig. 2A shows results for *CHS*1 and *V-ATPase*), by symptom development and by GFP fluorescence under UV light (for JL36-infected plants). These analyses identified recombinant TMVs containing *actin* RNA in the sense orientation (TMV-Actin), *CHS*1 RNA in sense (TMV-CHS1-S) and antisense (TMV-CHS1-AS) forms, and *V-ATPase* RNA in sense (TMV-V-ATPase-S) and antisense (TMV-V-ATPase-AS) forms. We further tested for siRNAs specific to the *CHS*1 RNA sequences by performing small RNA blot hybridization. RNAs from plants that were positive by RT-PCR also showed accumulation of the specific siRNAs (Fig. 2B).

### Feeding on plants infected with recombinant TMVs induces RNAi in *P. citri.*

**Figure 3:**
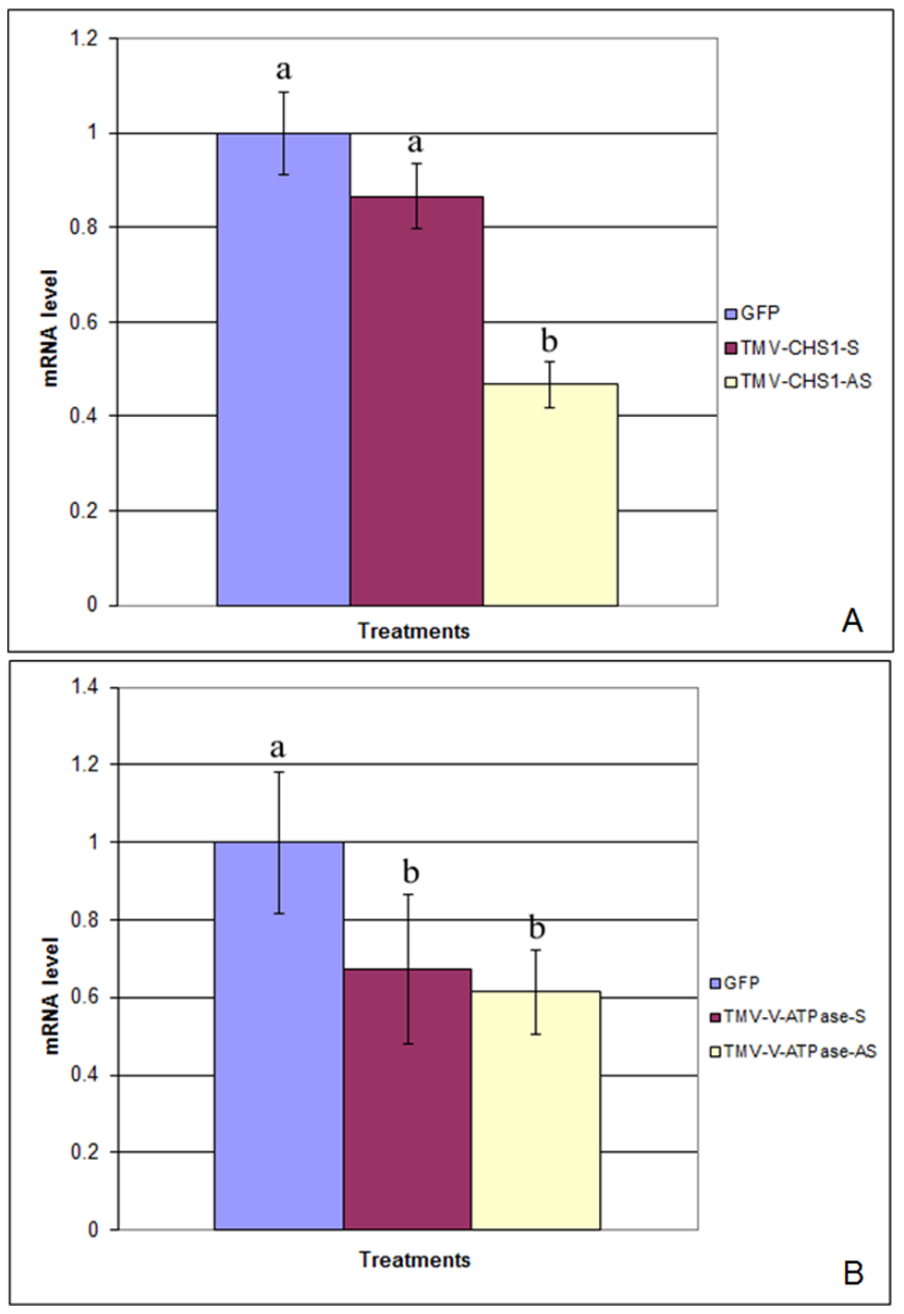
Relative levels of *CHS*1 (A) and *V-ATPase* (B) mRNAs in *P. citri* after feeding on *N. benthamiana* plants inoculated with recombinant TMV expressing *P. citri CHS*1 and *V-ATPase* fragments. qRT-PCR results of *CHS*1 (A) and *V-ATPase* (B) mRNA levels from *P. citri* after feeding on *N. benthamiana* plants infected with recombinant TMVs. TMV-GFP (pJL24) was used as a control and sense and antisense inserts of *P. citri CHS*1*/V-ATPase* sequences were compared. Total RNA was extracted after 12 days feeding on plants and used for cDNA synthesis. qRT-PCR was performed using primers described in Table 3. The 18S ribosomal RNA was used as an endogenous control in all experiments. Calculation of mRNA levels between control and treated groups was carried out by the comparative CT or 2^–ΔΔ^Ct method and analyzed by ANOVA using statistix 8.1 software. Numbers with the same letter indicate homogenous groups at p<0.01 (LSD 0.04 and 0.21) for *CHS*1 and *V-ATPase* respectively. All experiments were performed twice and data were used collectively.

After 12 days of continuous feeding on plants infected with the recombinant TMVs, we collected *P. citri* for RNA extraction and qRT-PCR analyses. These analyses showed that there was ∼10% reduction of the *P. citri CHS*1 mRNA (Fig. 3A) for *P. citri* fed on plants infected with the TMV-CHS1-S and a 50% reduction for *P. citri* fed on plants infected with TMV-CHS1-AS. There was a significant difference among *P. citri* fed on control plants infected with TMV-GFP, and those infected with TMV-CHS1-AS with an LSD value of 0.04 (P<0.01). Similarly ∼30–40% reduction was seen for the *V-ATPase* mRNA (Fig. 3B) for *P. citri* fed on plants infected with both TMV-V-ATPase-S and TMV-V-ATPase-AS vs. those fed on plants infected with TMV-GFP, with an LSD value of 0.21 (P<0.01).

### Reduction in *P. citri actin* mRNA correlates with reduced fecundity on recombinant TMV-Actin infected *N. benthamiana* plants

**Figure 4:**
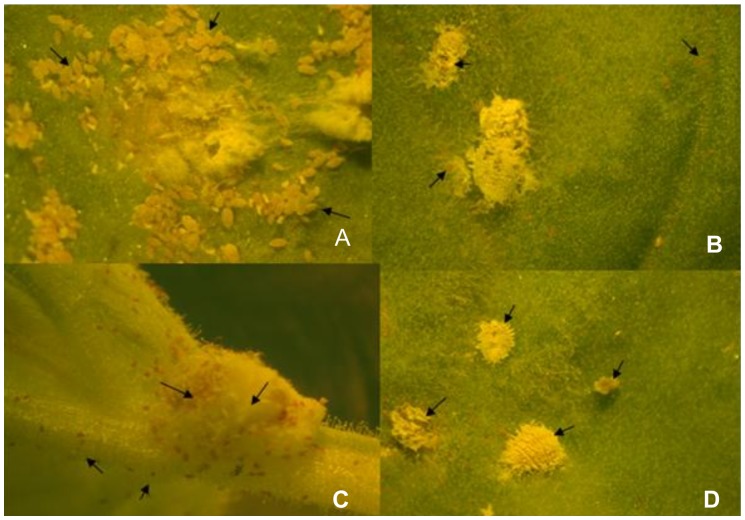
*P. citri* feeding on *N. benthamiana* plants inoculated with TMV-Actin. Mealybug crawlers feeding on: A) *N. benthamiana* plants inoculated with TMV only (pJL36) showing healthy crawlers emerging while B, C and D show *N. benthamiana* plants inoculated with recombinant TMV (TMV-Actin) and show a very high mortality in crawlers and adults. Arrows in A indicate healthy crawlers; in B & C arrows indicate dead crawlers and D the arrows indicate dead adults.

**Figure 5:**
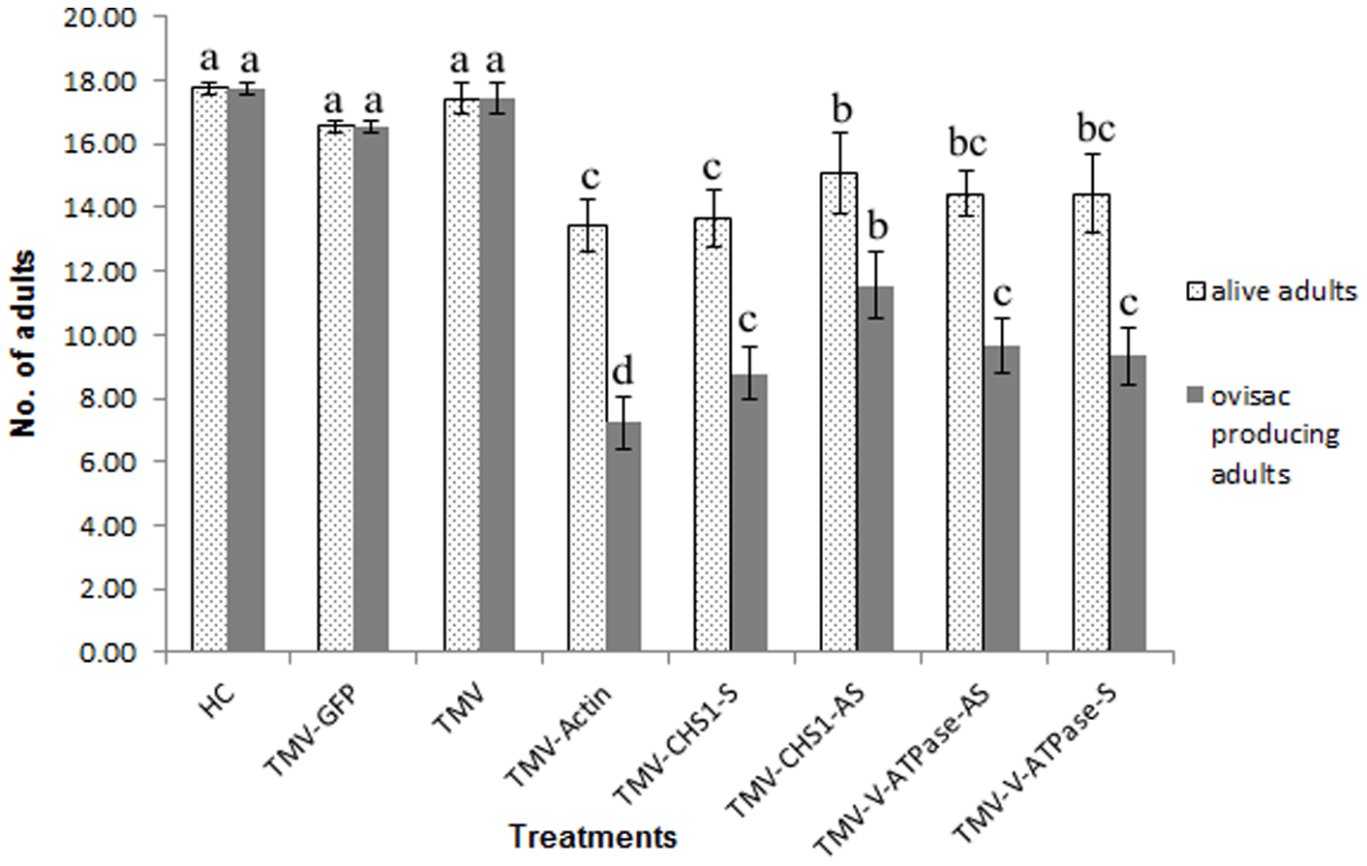
Ovisac production and survival of adult *P. citri* fed on plants inoculated with different recombinant TMV constructs. Number of surviving adult *P. citri* and ovisac production following 12 days feeding on plants inoculated with different recombinant TMV constructs. HC  =  healthy control *N. benthamiana* plants; TMV-GFP  =  TMV carrying GFP (TMV JL24); TMV  =  TMV with no insert (pJL36); TMV-Actin  =  part of the *actin* RNA inserted into TMV; TMV-CHS1-S and TMV-CHS1-AS  =  the *chitin synthase* 1 (*CHS*1) RNA fragment inserted into TMV in sense and antisense orientations, respectively; TMV-V-ATPase-S and TMV-V-ATPase-AS  =  the *V-ATPase* RNA fragment inserted into TMV in sense and antisense orientations, respectively. Numbers indicated with the same letter are homogenous groups at p<0.05.

**Figure 6:**
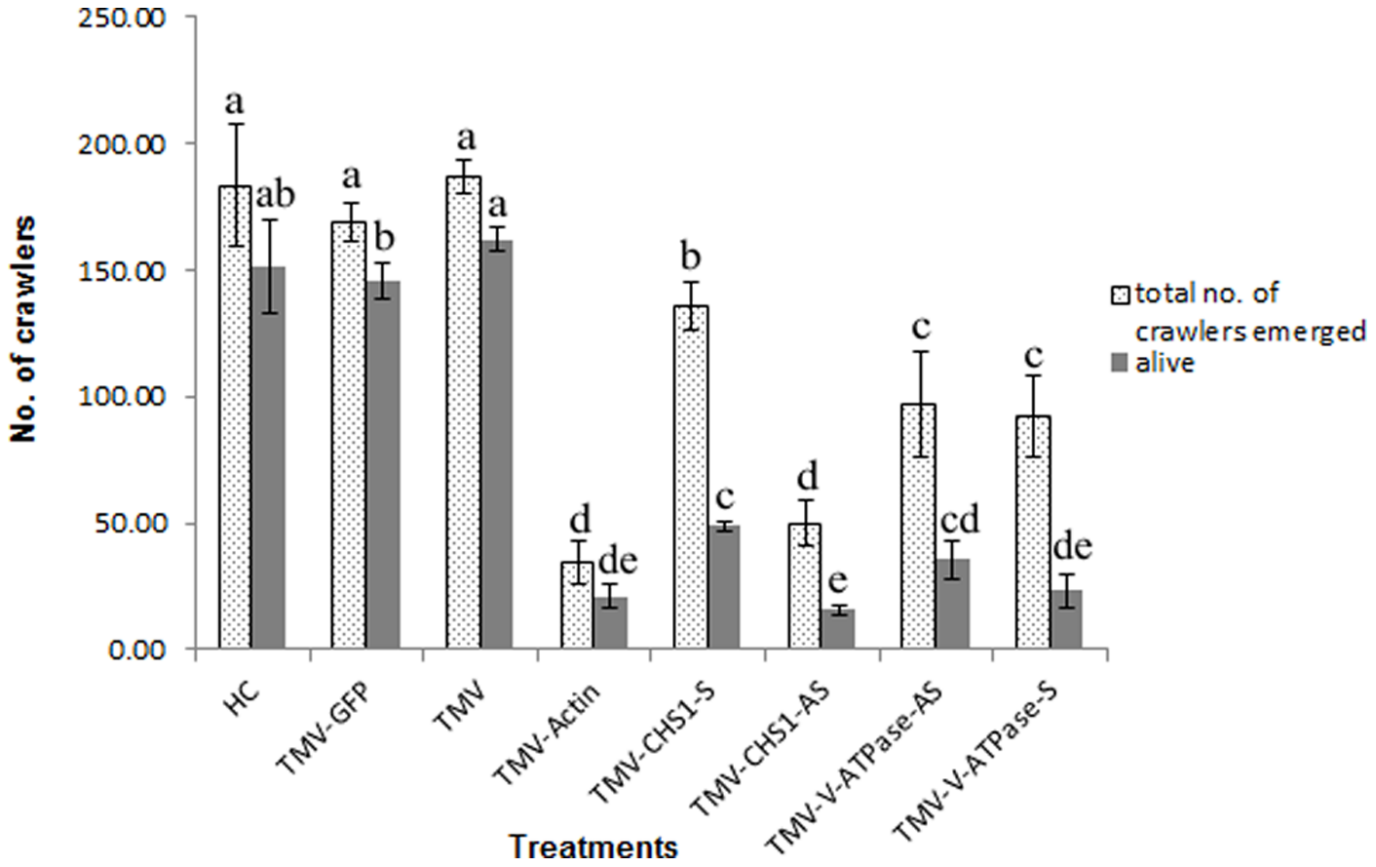
Emergence of *P. citri* crawlers and their survival on plants inoculated with different recombinant TMV constructs. Comparison of alive and total crawlers that emerged 18*P. citri* onto plants inoculated with the following viral constructs: HC  =  healthy control *N. benthamiana* plants; TMV-GFP  =  TMV carrying GFP (TMV JL24); TMV  =  TMV with no insert (pJL36); TMV-Actin  =  part of the *actin* RNA inserted into TMV; TMV-CHS1-S and TMV-CHS1-AS  =  the *chitin synthase* 1 (*CHS*1) RNA fragment inserted into TMV in sense and antisense orientations, respectively; TMV-V-ATPase-S and TMV-V-ATPase-AS  =  the *V-ATPase* RNA fragment inserted into TMV in sense and antisense orientations, respectively. Numbers indicated with the same letter are homogenous groups at p<0.05.

We observed that after continuous feeding on plants infected with recombinant TMV-Actin, there was no significant difference (P<0.05, LSD: 1.29) in survival of adult *P. citri* on all three control treatments: healthy plants, plants inoculated with TMV-GFP and plants inoculated with TMV only, showing mean survival of 17.78±0.19, 16.56±0.19 and 17.44±0.51, respectively. However, there was a significant difference between control groups and *P. citri* fed on TMV-Actin infected plants showing mean survival of 13.44±0.84. Similarly, there was a significant difference (P<0.05, LSD: 1.40) in the number of adults producing ovisacs for *P. citri* fed on healthy plants, plants inoculated with TMV-GFP, or on TMV-inoculated plants as compared to plants infected with TMV-Actin showing the mean number of adults producing ovisacs as 17.78±0.19, 16.56±0.19, 17.44±0.51 and 7.22±0.84 respectively (Figs. 4 & 5). Furthermore, crawlers emerging from ovisacs on TMV-Actin infected plants were greatly reduced (P<0.05, LSD: 25.69), with the mean number of emerging crawlers 34.44±8.39 (Fig. 4 & 6) as compared to the number of crawlers emerging from ovisacs on healthy control plants (183.33±24.04), on plants infected with TMV-GFP (168.89±7.70) and on plants that were infected with TMV only (186.67±6.67). There was no significant difference among all three control groups with respect to adult mortality, ovisac production, or crawler emergence and mortality in crawlers.

### Decrease in mRNA level of *CHS*1 and *V-ATPase* correlates with decrease in fecundity and increased mortality of *P. citri* crawlers

**Figure 7:**
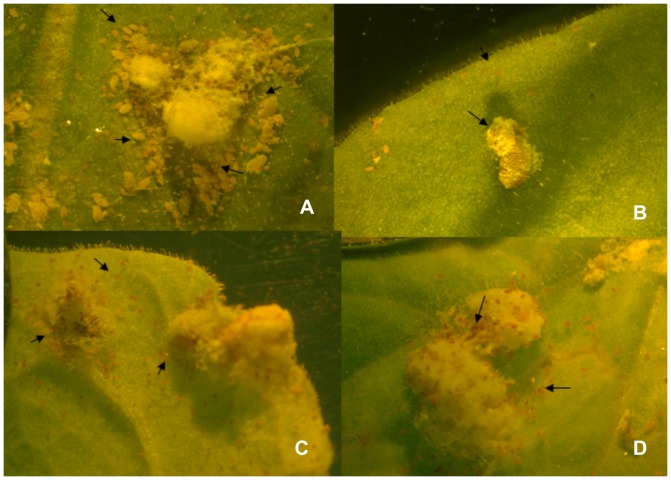
*P. citri* feeding on *N. benthamiana* plants inoculated with TMV-CHS1 and TMV-V-ATPase. Mealybug crawlers feeding on: A) *N. benthamiana* plants infected with TMV (pJL36) show healthy crawlers emerging while B and C show *N. benthamiana* plants infected with TMV-CHS1-S where B shows abnormal ovisac formation and C shows a high mortality of crawlers. D shows an *N. benthamiana* plant infected with TMV-V-ATPase-S showing a high mortality in crawlers. Arrows in A indicate healthy crawlers, in B an abnormal ovisac and in C & D indicate dead crawlers.

**Figure 8:**
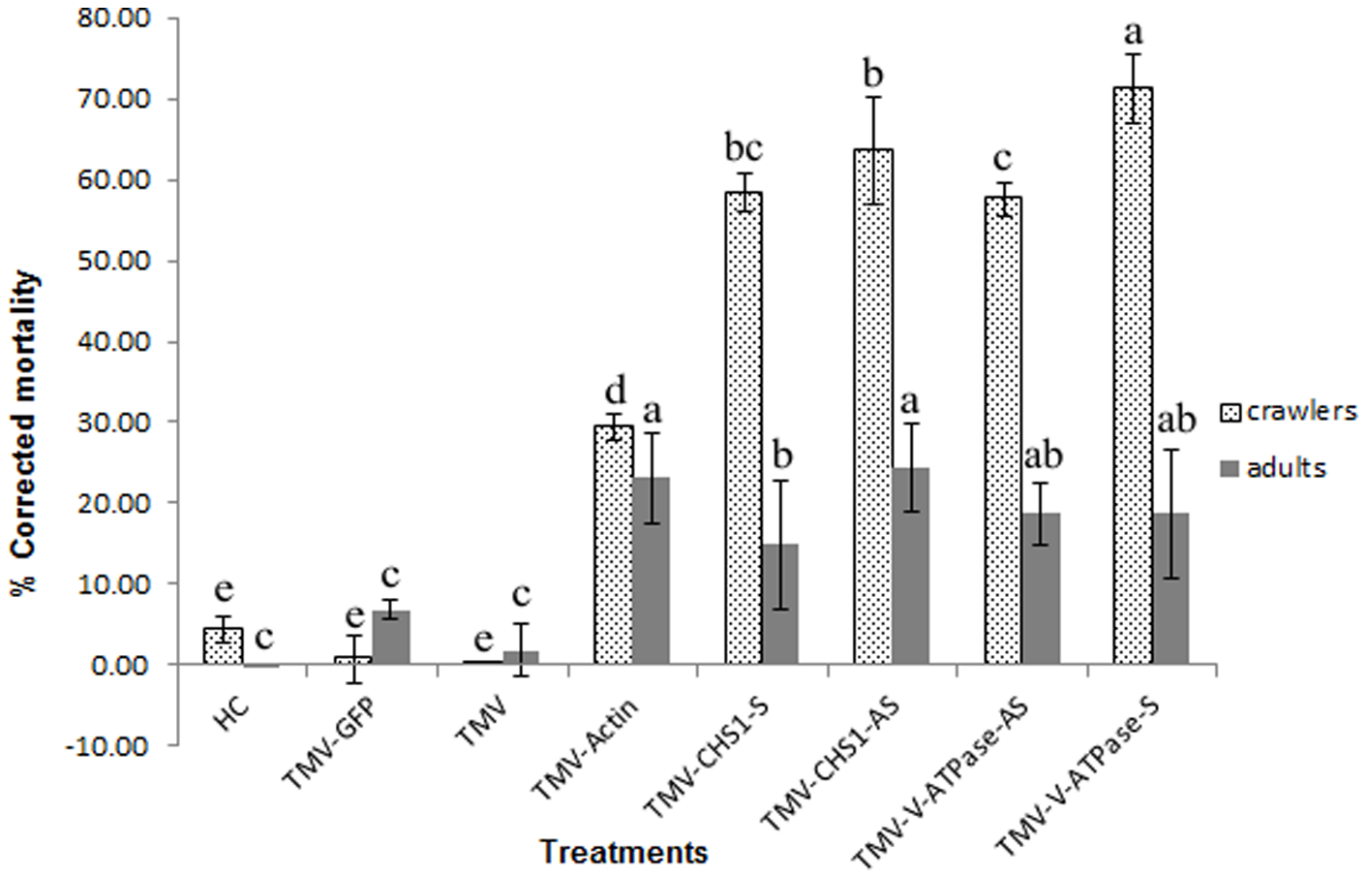
Mortality in *P. citri* adults and crawlers on plants inoculated with different recombinant TMV constructs. Comparison of corrected mortality in adult *P. citri* and crawlers following 18 days after release of insects onto plants inoculated with the following viral constructs: HC: healthy control *N. benthamiana* plant; TMV-GFP  =  TMV carrying GFP (TMV JL24); TMV  =  TMV with no insert (pJL36); TMV-Actin  =  part of the *actin* RNA inserted into TMV; TMV-CHS1-S and TMV-CHS1-AS  =  the *chitin synthase* 1 (*CHS*1) RNA fragment inserted into TMV in sense and antisense orientations, respectively; TMV-V-ATPase-S and TMV-V-ATPase-AS  =  the *V-ATPase* RNA fragment inserted into TMV in sense and antisense orientations, respectively. Numbers indicated with the same letter are homogenous groups at p<0.05.

We observed that *P. citri* fed on plants infected with recombinant TMVs expressing the *CHS*1 and *V-ATPase* RNAs reached adult stage. The number of *P. citri* crawlers emerging from ovisacs and their survival when adults fed on plants infected with these recombinant TMVs were greatly reduced and significantly different from that seen for controls (Figs. 6 & 7). We also found that the mortality among *P. citri* placed on plants at the beginning of the bioassays were significantly lower than the mortality in the crawlers which emerged from the ovisacs of these parents (Fig. 8). Significant differences (P<0.05, LSD: 1.29) were also observed in survival of adult *P. citri* fed on plants infected with TMV-Actin, TMV-CHS1-S, TMV-CHS1-AS, TMV-V-ATPase-S, and TMV-V-ATPase-AS compared to the control groups. For *P. citri* fed on plants infected with TMV expressing *CHS*1 and *V-ATPase* RNA fragments there was a significant difference (P<0.05, LSD: 1.40) between the control and treated groups for ovisac production (TMV-CHS1-S 8.78±0.84, TMV-CHS1-AS 11.56±1.07, TMV-V-ATPase-S 9.33±0.88 and TMV-V-ATPase-AS 9.67±0.88). There was no significant difference between all three control groups, and similarly no significant difference between plants infected with TMV expressing sense and antisense *V-ATPase* sequences, but ovisac production was significantly lower in adults fed on plants infected with TMV-CHS1-S as compared to plants infected with TMV-CHS1-AS. A significant difference (P<0.05, LSD: 5.75) was seen between control groups (healthy plants 4.48±1.63, plants inoculated with TMV-GFP 0.82±2.94 and in plants inoculated with TMV only 0.00±0.00) and plants infected with the recombinant TMVs containing *CHS*1 and *V-ATPase* RNA sequences for mortality in crawlers showing mean corrected mortality of 58.41±2.39 and 63.66±6.63 for TMV-CHS1-S and TMV-CHS1-AS, respectively, and 71.22±4.22 and 57.67±2.07 for TMV-V-ATPase-S and TMV-V-ATPase-AS, respectively (Figs. 7 & 8).

## Discussion

We first demonstrated RNAi effects in *P. citri* after injection of specific dsRNAs. This approach has been utilized by researchers [37], [38] on different insects for screening candidate targets for RNAi, but there is often high variability in terms of insect mortality due to the injection procedures, target RNAs and the target insect. Furthermore, evaluating RNAi effects for practical potential often does not reflect reality as injection is an artificial delivery system. For phloem-feeding hemipterans, such as *P. citri*, practical delivery is likely to be via the plants upon which the target insects feed. In order to achieve *in planta* evaluations of potential RNAi effectors, we used the recombinant virus-based vector, TMV. We hypothesized that plant viruses can be effective tools for targeting plant-feeding hemipterans by RNAi as viruses are both targets and powerful inducers of RNAi activity in plants [39]. Recently, *Tobacco rattle virus* (TRV) expression of antisense fragments from a chewing insect, *M. sexta,* in *Nicotiana attenuata* plants specifically silenced three midgut-expressed *MsCYPs* RNAs when larvae fed on these plants [32], and recombinant TMV was used to induce RNAi effects in *Bactericera cockerelli* on tomatillo plants [40]. Our data and that of Wuriyanghan and Falk [40] suggest that RNAi effectors can be present in the phloem in sufficient amounts to induce RNAi activity in phloem feeders, and other analyses have shown that siRNAs can be recovered from phloem sap [16].

We used the plant, *N. benthamiana,* due to its susceptibility to both the TMV and *P. citri*. We first confirmed by RT-PCR that the *P. citri* mRNA sequences were retained by recombinant TMV in infected plants and we used small RNA hybridization to demonstrate the presence of specific *P. citri* siRNAs in recombinant virus-infected plants. When *P. citri* were fed on *N. benthamiana* plants infected with recombinant TMV, we were able to detect specific RNAi effects including target mRNA reductions, *P. citri* mortality and reduced fecundity and hampered growth. Interestingly, the RNAi effects were much more prominent in the emerging nymphs/crawlers than in the adults which were initially placed to the TMV-inoculated plants. Wuriyanghan and Falk [40] also noted phenotypic effects on nymphs of the phloem-feeding *B. cockerelli*. Our combined data suggest that these effects may be due to the fact that the developing nymphs feed more voraciously than the older adults, hence can take up greater amounts of RNAi inducers as compared to adults, or they may be better RNAi targets.

Our qRT-PCR data showed significant reductions of the target mRNAs. Both dsRNA injection experiments and when *P. citri* were fed recombinant TMV-infected plants showed reductions in the corresponding mRNA target. By contrast, injection of GFP dsRNAs, or when *P. citri* fed on TMV-GFP-infected plants target mRNA reductions were not reduced. We cannot be sure that some non-target mRNAs were not also reduced (off target effects), but as the mRNAs evaluated here were not affected by GFP effectors and as we designed our sequences carefully using “SnapDragon” software (http://www.flyrnai.org/cgi-bin/RNAi_find_primers.pl), our data suggest that target mRNA reductions seen here were due to RNAi.

We also observed high mortality in adult mealybugs on plants infected with TMV-Actin and significant mortality (P<0.05) also on plants infected with TMV-CHS1 and TMV-V-ATPase. Interestingly we observed high mortality of 71.22±4.22 in the emerging *P. citri* crawlers on plants infected with TMV-V-ATPase. Crawler emergence and mortality were significantly different between samples fed on control group plants and TMV-V-ATPase infected plants. For *P. citri* fed on plants infected with TMV-CHS1, we found significant differences for plants infected with recombinant TMV having *CHS*1 inserts in the sense (TMV-CHS1-S) vs. antisense (TMV-CHS1-AS) orientations with respect to crawler emergence and mortality. Similar trends were also observed in qRT-PCR results where there was a significant difference in sense and antisense only for *CHS*1 constructs.

Previous studies have shown successful RNAi effects in insects through artificial feeding of siRNAs/dsRNAs, and through transient or stable transformation of plants expressing interfering RNAs [15]-[17], [19], [37], [41]–[43]. Our studies here provide another direction for rapid, yet functional *in planta* RNAi studies. TMV is easily manipulated, has a wide plant host range and gives fairly quick effects in plants. This approach provides a robust method to study the RNAi effects towards plant feeding insects, even phloem feeders, and the dramatic effects seen here by us particularly on nymphal/carwler mealybugs gives strong support for further efforts in RNAi-based insect control.

## Supporting Information

Tables S1 **Includes Table S1- Table S3.**
(DOCX)
