# Supplementary material for: Use of Recombinant Tobacco Mosaic Virus To Achieve RNA Interference in Plants against the Citrus Mealybug, Planococcus citri (Hemiptera: Pseudococcidae)
Source: PLoS One. 2013 Sep 9;8(9):e73657. doi: 10.1371/journal.pone.0073657 (PMC3767618; doi:10.1371/journal.pone.0073657)
Supplement: Tables S1 — Includes Table S1- Table S3. (DOCX) [file pone.0073657.s001.docx]

Supplementary Tables 1 - 3

**Table S1: Comparison of Nucleotide Sequence of the *Planococcus citri* *CHS*1 gene with those of the *CHS*1 gene sequences from other insects as determined by NCBI Blast Search.**

**Species Nucleotide identity (%) Accession number**

*Phenacoccus solenopsis* 87 unpublished

*Lucilia* *cuprina* 80 [AF221067.1](http://www.ncbi.nlm.nih.gov/nucleotide/9963822?report=genbank&log$=nucltop&blast_rank=1&RID=SKZPHKMJ01R)

*Aphis glycines* 79 [JQ246352.1](http://www.ncbi.nlm.nih.gov/nucleotide/386266702?report=genbank&log$=nucltop&blast_rank=6&RID=SM56A76P01R)

*Lucilia sericata* 79 [EF056212.1](http://www.ncbi.nlm.nih.gov/nucleotide/117668124?report=genbank&log$=nucltop&blast_rank=7&RID=SM56A76P01R)

*Anasa tristis* 79 [JQ398680.1](http://www.ncbi.nlm.nih.gov/nucleotide/391226610?report=genbank&log$=nucltop&blast_rank=8&RID=SM56A76P01R)

*Locusta migratoria* 79 [GU067730.1](http://www.ncbi.nlm.nih.gov/nucleotide/262232648?report=genbank&log$=nucltop&blast_rank=10&RID=SM56A76P01R)

*Manduca sexta* 77 [AY062175.2](http://www.ncbi.nlm.nih.gov/nucleotide/24762311?report=genbank&log$=nucltop&blast_rank=21&RID=SM56A76P01R)

*Choristoneura fumiferana* 77 [EU561238.1](http://www.ncbi.nlm.nih.gov/nucleotide/189212450?report=genbank&log$=nucltop&blast_rank=23&RID=SM56A76P01R)

*Nilaparvata lugens* 76 [JQ040014.1](http://www.ncbi.nlm.nih.gov/nucleotide/378830223?report=genbank&log$=nucltop&blast_rank=38&RID=SM56A76P01R)

*Tribolium castaneum* 74 [NM_001039402.1](http://www.ncbi.nlm.nih.gov/nucleotide/86515337?report=genbank&log$=nucltop&blast_rank=61&RID=SM56A76P01R)

**Table S2: Comparison of Nucleotide Sequence of the *Planococcus citri V-ATPase* gene with those of the *V-ATPase* gene sequences from other insects as determined by NCBI Blast Search.**

**Species Nucleotide identity (%) Accession number**

*Phenacoccus solenopsis* 83 unpublished

*Bombus impatiens* 78 [XM_003492267.1](http://www.ncbi.nlm.nih.gov/nucleotide/350419828?report=genbank&log$=nucltop&blast_rank=1&RID=SKVX7KR6015)

*Bombus terrestris* 77 [XM_003397802.1](http://www.ncbi.nlm.nih.gov/nucleotide/340718797?report=genbank&log$=nucltop&blast_rank=2&RID=SKVX7KR6015)

[*Drosophila willistoni*](http://blast.ncbi.nlm.nih.gov/Blast.cgi#alnHdr_195434355) 77 [XM_002065133.1](http://www.ncbi.nlm.nih.gov/nucleotide/195434355?report=genbank&log$=nucltop&blast_rank=3&RID=SKVX7KR6015)

*Aedes aegypti* 76 [AF008922.1](http://www.ncbi.nlm.nih.gov/nucleotide/2454487?report=genbank&log$=nucltop&blast_rank=9&RID=SKVX7KR6015)

*Aedes* *albopictus* 76 [AY864912.1](http://www.ncbi.nlm.nih.gov/nucleotide/61971316?report=genbank&log$=nucltop&blast_rank=1&RID=SKYYRNXE01R)

**Table S3: Comparison of Nucleotide Sequence of the *Planococcus citri* *β-Actin* gene with those of the *β-Actin* gene sequence from other insects as determined by NCBI Blast Search.**

**Species Nucleotide identity (%) Accession number**

*Phenacoccus solenopsis*  88 unpublished

*Planococcus* *citri* 87 [JX068849.1](http://www.ncbi.nlm.nih.gov/nucleotide/399207751?report=genbank&log$=nucltop&blast_rank=1&RID=SM1EU0XS015)

*Bombus* *impatiens* 85 [XM_003486524.1](http://www.ncbi.nlm.nih.gov/nucleotide/350402701?report=genbank&log$=nucltop&blast_rank=2&RID=SM1EU0XS015)

*Apis* *cerana* 84 [JX899419.1](http://www.ncbi.nlm.nih.gov/nucleotide/432141057?report=genbank&log$=nucltop&blast_rank=6&RID=SM1EU0XS015)

*Megachile* *rotundata* 83 [AY550118.1](http://www.ncbi.nlm.nih.gov/nucleotide/45331062?report=genbank&log$=nucltop&blast_rank=11&RID=SM1EU0XS015)

*Liposcelis* *entomophila* 76 [FJ041117.1](http://www.ncbi.nlm.nih.gov/nucleotide/199582624?report=genbank&log$=nucltop&blast_rank=35&RID=SM1EU0XS015)
